# Regular Physical Activity Seems to Eliminate Lower Limb Perfusion Asymmetries in Sedentary Non-Healthy Older Individuals

**DOI:** 10.3390/life14101258

**Published:** 2024-10-02

**Authors:** Margarida Florindo, João Gregório, Luís Monteiro Rodrigues

**Affiliations:** 1 CBIOS—Research Center for Biosciences and Health Technologies, Universidade Lusófona, Av. Campo Grande, 376, 1749-024 Lisboa, Portugal; joao.gregorio@ulusofona.pt (J.G.); monteiro.rodrigues@ulusofona.pt (L.M.R.); 2 ESSCVP—Department of Physiotherapy, The Portuguese Red Cross Health School, Av. De Ceuta, 1350-125 Lisbon, Portugal

**Keywords:** perfusion asymmetries, elderly, skin microcirculation, physical activity, cardiovascular

## Abstract

Perfusion asymmetries have been associated with vascular pathology. Physical activity is a component of vascular health. Here, we investigate the impact of a thirty-day regular home-based physical activity program in older individuals with multiple age-related comorbidities. Eleven participants of both sexes were included. Skin perfusion was assessed in both feet by laser Doppler flowmetry (LDF, for deeper perfusion) and polarized light spectroscopy (PSp, for superficial perfusion). At baseline, participants had significant perfusion differences between right and left feet (*p* = 0.005 with LDF; *p* = 0.028 with PSp), which disappeared with activity and reappeared in recovery. After 30 days, both feet showed significant perfusion increases as assessed with LDF (*p* = 0.005) compared to D0. At this point, the perfusion asymmetry observed at D0 was no longer present. However, the superficial perfusion asymmetry assessed by PSp persisted at D30 (*p* = 0.028). Data show that regular physical activity positively altered the lower limb perfusion pattern. Systolic pressure and mean arterial pressure were also significantly reduced at D30. These impacts seem to be directly related to the physical activity program introduced in the day-by-day routines of these participants. These also encourage further research on the role of symmetry–asymmetry in prevention, treatment, and rehabilitation in vascular medicine.

## 1. Introduction

Lower limb perfusion asymmetries, perceived as differences in resting baseline perfusion between paired limbs in healthy individuals, are known but their significance has only been recently discussed [1,2]. Discussion on this theme was first registered at the end of the last century following data analysis from nuclear magnetic resonance flowmetry [3], where significant asymmetries in lower limb arterial disease patients were identified. The same study highlighted significant correlations between asymmetric paired legs in healthy patients.

The physiological meaning of these asymmetries has not been sufficiently explored. Being mostly accepted as a result of anatomical unevenness involving major vessels such as the radial, mesenteric, femoral, and popliteal arteries, only a few papers approached these differences—particularly in the absence of disease—in the last few years [1,4,5,6]. However, the data are still insufficient and inconclusive. Perfusion asymmetries between paired limbs have also been associated with an increased risk of injury in high-performance sports but recent reviews confirmed that most of the data and findings are not consistent [7,8].

Part of our team’s expertise has been dedicated to the study of microcirculatory dynamics and adaptive homeostatic responses to distal changes in perfusion. This research has led to the identification of baseline perfusion asymmetries in healthy cohorts of both sexes and different ages, which, once submitted to various challenges, disappeared to reappear during recovery. We noticed that activities such as bipodal plantar flexion or walking would naturally produce effects in both limbs’ perfusions [9,10,11]. Similar observations were also present in both limbs when the challenge was applied in one of the legs, such as with massage [11,12,13] or the unipodal squat/hemi-squat [14]. We also noticed that perfusion asymmetries were more visible in older healthy participants, where a slower recovery after the challenge was noted [1]. Recent publications underlined the importance of shear stress, turbulence, and pulsatility as major determinants of microcirculatory pathology, suggesting that these might potentially determine or influence disease progression in distal territories such as in the lower limb in predisposed individuals [6,15,16,17]. Therefore, we considered these perfusion asymmetries might be an early indicator of cardiovascular impairment. Furthermore, since all of the challenges we used to explore perfusion adaptive mechanisms eliminated perfusion asymmetries between limbs for a certain period of time, we also thought that regular physical activity might impact and potentially improve distal perfusion and global hemodynamics in sedentary individuals. The importance of physical activity and exercise in cardiovascular health is well-established [17], but systematic reviews and meta-analyses regarding the effects of regular physical activity or exercise on specific cardiovascular variables, especially in older non-healthy populations, are very limited or even absent [18].

Therefore, the present study was primarily conceived following these recent observations involving lower limb perfusion asymmetries in the absence of occlusive arterial disease, identifying these as an early indicator of impairment. This study involved regular light physical activity and was implemented by a home-health program inserted into all participants’ daily routines. The participants comprised a non-healthy cohort of sedentary older people with several age-related comorbidities. More than contributing to knowledge on the significance of these lower limb perfusion asymmetries in the absence of vascular disease, we also suggest the possibility of using perfusion symmetry-based research to better understand cardiovascular pathophysiology.

## 2. Materials and Methods

### 2.1. Sample

A convenience sample involving eleven participants of both sexes (five women, mean 62.6 ± 8.3 years old, and six men, mean 62.2 ± 1.3 years old) was selected following specific inclusion criteria. Considering the study’s exploratory nature, the sample size calculation was regarded as unnecessary. Inclusion criteria required being over 50 years old, showing one or more comorbidities associated with age (obesity, hypertension, dyslipidemia, and/or diabetes) but no vascular disease, with a sedentary lifestyle (assessed through the number of minutes of physical activity per week and the number of steps counted by mobile phone) [17]. Non-inclusion criteria included an altered Ankle–Brachial Index (ABI) value, an important clinical indicator of the presence of occlusive arterial disease of the lower limbs in symptomatic and non-symptomatic individuals [19,20], and any type of compromise in lower limb mobility and gait (Table 1). Participants were required to have a smartphone and be familiar with the regular use of the application “Google Fit” for registering their active minutes every day. From these, we calculated the minutes of physical activity per week for each participant on Day “0” (D0) (Table 1).

### 2.2. Experimental Procedures and Variables

The procedures respected all principles of good clinical practice recommended by the World Medical Association’s Declaration of Helsinki and respective amendments [21], which included an informed written consent form signed by all participants before starting the study. The study protocol was previously approved by the Institutional Ethics Committee for Health Sciences (EC.ECTS/P03.20).

All measurements were conducted in our laboratories by the same researcher.

After completing the inclusion actions involving the detailed explanation of the study objectives and procedures; a period of adaptation to the laboratory conditions (20 to 30 min; temperature, 24.0 ± 1.0 °C; humidity, 54.0 ± 2.0%; and reduced light intensity) was observed. Brachial and ankle blood pressures were measured using a digital sphygmomanometer (Pic 22012000200 Sphygm Classic Check; Artsana S.p.A., Como, Italy); with the participant in dorsal decubitus to calculate the ABI.

This initial session (D0) was also used to demonstrate to each participant how to perform each activity at home—using live demonstrations and a schematic diagram—and to clarify any doubts.

**Figure 1:**
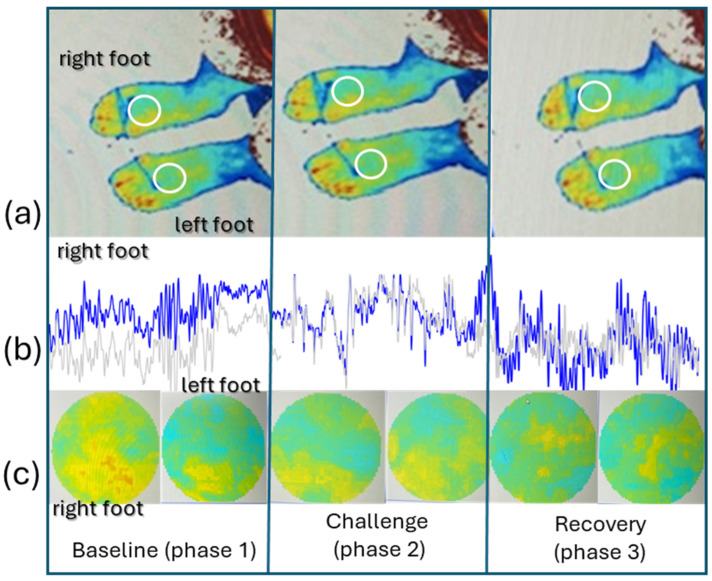
Illustrative example of PSp perfusion recordings during the different phases of the experimental protocol, in one participant, at D0, showing (**a**) the regions of interest (ROI) selected in both feet, (**b**) the perfusion line graph obtained from each foot by D0, and (**c**) the corresponding color contrast images, which represent different light absorption from red blood cells in both feet. As visible in these images, the perfusion asymmetries noted at rest disappear after the challenge during recovery (see text).

After a brief period of postural adaptation (10 min) in the upright position, systolic pressure, diastolic pressure, mean arterial pressure, pulse rate, and perfusion were registered. Perfusion was measured in both feet using laser Doppler flowmetry (LDF) and polarized spectroscopy (PSp). These two optical-based technologies use different light frequencies to interact with the tissue, providing different in-depth readings according to their penetration and dispersion properties [22,23]. LDF probes (Perimed PF5010 System; Perimed, Stockholm, Sweden) were placed perpendicular to the surface of the skin in the fourth metatarsophalangeal joint of both feet. This contact technology is accepted as a reference method for continuous perfusion assessment of red blood cells’ movement in a single location at a depth of 0.3 to 1 mm [22,23]. The PSp system (Tissue Viability Image System-TiVi701; WheelsBridge, Linkoping, Sweden) was used to simultaneously obtain perfusion images at the skin surface. PSp is a non-contact technology using a digital camera coupled with parallel and perpendicular polarized filters. Images were taken at 30 cm, with both feet resting horizontally on a green background plate. Digitalization produces a pseudo-colored image as the result of an algorithm sensitive to the concentration of red blood cells in the skin’s superficial vascular plexus [24,25]. A region of interest (ROI) measuring approximately 15 mm in the center of the dorsum of each foot was selected for evaluation (Figure 1).

At day zero (D0), duly informed participants followed a prescribed light physical activity program guided by the FITT principle (F—frequency, I—intensity, T—time/duration, and T—type of activity) [26] and designed to be followed at home daily for 30 days. The frequency (F) was set at once a day considering each participant’s daily physical activity calculation (previously assessed with the help of the “Google Fit” application on their smartphones); the program’s intensity (I) was set by exposing all participants to the specified sequence of movements involving five minutes of ”step in place” at a comfortable speed, chosen by the participant; one minute of isometric plantar flexion; and five minutes of walking also at a comfortable pace. This exercise series added 11 min/day (77 min/week) to each participant’s daily physical activity, contributing to reaching the WHO recommended minimum of 150 min of activity per week. The intensity of these activities was assessed by the Karvonen formula [(Max Heart Rate-Resting Heart Rate) × 60% + Resting Heart Rate] [26], confirming that the program could be classified as a light activity. The protocol time (T) was defined for four weeks to ensure intrinsic functional and vascular changes at foot level (28–30). We structured this program according to a sequence of activity types (T) that were previously studied and influenced the perfusion of the foot [12,13,14,15,16]. D0 was also the recording day for the baseline data. During the course of the program, the investigator confirmed by weekly video chat that each participant was compliant with the protocol previewed conditions. By D30, the minutes of activity were also registered and compared with D0.

The experimental design included three phases: phase 1, corresponding to the register of basal conditions with volunteers standing for 5 min; phase 2, where recordings were taken after the program completion; and phase 3, corresponding to the recovery register, with participants standing for five minutes. These measurements were repeated on day 30 (D30), after finalizing the program, and compared with D0.

### 2.3. Statistical Procedures

Collected data were organized in Excel tables (Microsoft^®^ Excel^®^ for Microsoft 365 MSO, version 2306 Build 16.0.16529.20164) and analyzed by descriptive and comparative statistics using IBM SPSS Statistics for Windows software (Version 28.0. IBM Corp, Armonk, NY, USA). After testing the normal distribution of data for each limb, and considering the low number of participants, a decision was made to use only non-parametric tests in all variables. We used the Mann–Whitney non-parametric test for independent samples to assess perfusion asymmetries. We also explored the impact of the intervention between D0 and D30 using the non-parametric Wilcoxon test to compare the mean perfusion variations. A *p*-value of ≤0.05 for significance was adopted.

## 3. Results

**Figure 2:**
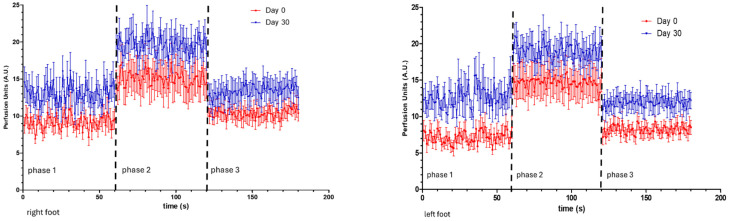
Real-time LDF perfusion register from all participants at D0 and D30 in both lower limbs (check text).

Significant distal perfusion asymmetries were detected at both measured skin depths in all participants at D0 (LDF, *p* = 0.005; PSp, *p* = 0.028, Figure 2, Table 2), with the right foot consistently showing higher perfusion values than the left foot. Potential dominance factors were not investigated. After performing the sequence of activities, perfusion asymmetries disappeared in the deeper structures (as measured by LDF) but remained in the upper vessels (PSp *p* = 0.022, Table 2). In the recovery period, asymmetries were again present in the deeper vessels (LDF, *p* = 0.017) but not in those more superficial (Table 2).

The number of minutes of activity registered by the smartphone application after 30 days of the activity program by D30 was significantly higher than the register at D0 (87.3 ± 12.7 and 205.8 ± 14.7 min, respectively). The baseline perfusion significantly changed in the deeper structures assessed by LDF. To illustrate these changes, we calculated the ratio D0/D30 for each technology. This ratio showed that perfusion in the deeper vessels increased in both feet, mainly in the left foot (76.4%) when compared with the right foot (42.4%), balancing distal perfusion at these points and causing the perfusion asymmetries to disappear. At the superficial vessels, however, asymmetries were still found in baseline measurements (phase 1) with PSp by D30. Consistently lower than D0 measurements, the calculated D0/D30 ratio showed 10.7% on the right and 4.9% on the left, resulting in a persisting significant asymmetry (*p* = 0.007, Table 2). By D30, no other asymmetries could be detected with either technology after performing the activity sequence or during recovery (Table 2). The D0/D30 ratio for each technology showed that after 30 days of these regular activities, the perfusion increase measured in phase 2 in both feet was balanced and consistent (for LDF, (+)31.3% and (+)30.1% on the right and left feet, respectively; for PSp, (+)6.6% and (+)9.3% on the right and left feet, respectively) as in phase 3 (for LDF (+)28.9% and (+)46.3% on the right and left feet, respectively; for PSp 6.9% and 6.7% on the right and left feet, respectively).

A significant reduction in other hemodynamic variables, such as systolic blood pressure (SYS_P) and mean arterial pressure (MAP), was registered at D30 (Table 3).

## 4. Discussion

Pathological asymmetries in the circulatory system have been known of since the first description of peripheral arterial disease and the identification of arterial blood pressure unevenness between upper and lower limbs in healthy individuals [27]. Morphological changes were primarily indicated as major determinants. Anatomical asymmetries were found in both the femoral and radial arteries of healthy individuals, and hemodynamic unevenness in the deep and superficial femoral arteries was observed, primarily related to the lower limb muscular function rather than morphology [4,6,28]. However, changes in vascular geometry were suggested to increase the levels of hemodynamic parameters, changing the blood flow and the wall shear stress, acting as determinants of local vascular injury [10,29,30,31]. Recent data suggest that the intracranial aneurysm distribution is associated with asymmetric vascular structures [32,33] and that asymmetries in paired vessels morphology, including branching and curvatures, seem to be linked to pathological processes such as atherosclerosis, arterial dissection, and aneurysms [34].

Clearly, perfusion asymmetries have been almost exclusively reported in large vessels, likely related to the connections already long-established with major processes such as peripheral arterial disease and athero- and arteriosclerosis [6,28,35,36,37]. However, our previous research was the first (to our knowledge) to describe physiological perfusion asymmetries in the distal microcirculation of healthy individuals and the role of determinants such as age, sex, and BMI [1,2]. Our results also highlighted the importance of hemodynamics on distal perfusion asymmetry by focusing on the impact of adaptive circulatory mechanisms, as previously cited. This outcome made the perfusion asymmetry easily quantifiable, eliminating subjective descriptions of its definition.

Our studies align with the inter-arm blood-pressure difference (IAD) [37], an indicator explored within the same conceptual basis. An inter-limb systolic blood difference of 10 mmHg in the arm and 15 mmHg in the leg was found to be related to the risk of cardiovascular disease and used as a surrogate marker for early renal impairment in patients with type 2 diabetes. However, our option was to add to the asymmetry assessment, finding an interplay between central and local circulatory regulation—also affecting global hemodynamics—as seen by a controlled change in local perfusion [35,38,39]. Our research indicated that this simple intervention, introducing a daily regular light intensity activity in the lower limb, could potentially reduce or eliminate perfusion asymmetries in these patients and affect eventual vulnerabilities related to the perfusion asymmetry, independent of its origin. Under this rationale, in line with the most recent findings regarding exercise and cardiovascular aging [18], we developed the present study, designed within a previously tested home-based program, to be applied in this non-healthy cohort of participants for 30 days with minimum supervision. Perfusion asymmetries were intentionally assessed by two optical technologies, having in mind the particular skin circulatory structure involving two vascular plexuses parallel to the skin at different depths: the deep plexus of larger vessels approximately located at 2–6 mm below the skin surface, and the superficial plexus involving smaller vessels placed approximately 0.3–2 mm below the epidermis [11,40]. Perpendicular structures connect both plexuses. As previously indicated, LDF uses a 780 nm red light, which interacts with deeper blood tissue (red blood cells), compared with the white light used by PSp, which only assesses the most superficial blood components.

As shown in Table 2, by D30, a notable increase in baseline LDF values is registered after the program application, more pronounced in the left foot when compared with the right foot (76.4% and 42.4%, respectively), such that the deeper perfusion is more symmetric. However, at D30, significant baseline asymmetries were still detected at the most superficial plexus, although values were lower than those obtained at D0 (Table 2). A more pronounced reduction was observed in the right foot compared with the left foot (10.7% and 4.9%, respectively). From this point on, the physical activity (phase 2) and recovery (phase 3) did not affect the perfusion in both limbs, unlike our observations on D0. These results should, in our opinion, be assumed as a direct consequence of the 30-day program of regular light-intensity physical activity, which was already suggested by the significant increase measured via LDF at the D30 baseline, compatible with higher muscular involvement [40]. Changes in the opposite direction at the most superficial areas—as measured by PSp—confirm this view, as a higher (hemodynamical) demand from the lower limb muscles will necessarily mobilize exchanges between the plexus, reducing the amount of blood at the surface during circulatory adaptation [40]. Also important, keeping in mind the comorbidity profile of these participants—half of whom used cardiovascular therapeutics for hypertension—it is noteworthy to register the significant reductions in SYS_P and MAP, both recognized as major determinants of cardiovascular risk. Finally, this pilot study draws attention to the consistent presence of perfusion asymmetries in the microcirculation of this cohort of older non-healthy individuals, in the absence of peripheral obstructive disease, in line with one of our hypotheses.

Recognized limitations are (i) the small number of participants and convenience sampling; (ii) the limited study duration; and (iii) the mild pathological profile of participants.

In conclusion, our study, even if exploratory, showed that regular activity caused these asymmetries to disappear, confirming the relevance of this topic to deepen our knowledge of vascular pathophysiology. More study models and data are needed to better understand the potential utility of perfusion asymmetry research in prevention, treatment, and rehabilitation in vascular medicine.

## Figures and Tables

**Table 1:** Global characterization of participants at inclusion (Day 0). Comorbidities and the related medications were identified. Results are presented as mean and standard deviation (mean ± sd). Main comorbidities present were pre-diabetes (50% of the participants), hypertension (50%), dyslipidemia (70%), and overweight (70%). Half of these participants presented two or more comorbidities.

Participants (n = 11)	Mean ± sd
Age, years old	62.4 ± 5.6
BMI, kg/m^2^	25.6 ± 2.9
ABI	1.1 ± 0.1
MAP, mmHg	95.7 ± 6.5
Steps/day (number)	3400.5 ± 826.7
Physical activity/week (min)	87.3 ± 12.7

BMI, body mass index; ABI, ankle–brachial index; MAP, mean arterial pressure.

**Table 2:** Perfusion recordings obtained at D0 before program initiation and at D30 after the program completion. Perfusion measurements obtained with optical technologies (LDF and PSp) in both right (R) and left (L) feet are shown for each of the experimental phases (1 to 3) reproduced at D0 and D30 (check text). (* Statistically significant differences.)

	**Phase 1 Baseline**			**Phase 2 Post-Activity**			**Phase 3 Recovery**		
	R0	L0	*p*	R0	L0	*p*	R0	L0	*p*
LDF PU	9.2 ± 2.8	7.2 ± 2.6	0.005 *	15.0 ± 7.1	14.6 ± 7.1	ns	10.4 ± 3.0	8.2 ± 2.5	0.017 *
PSp CRBC	125.4 ± 23.7	114.6 ± 19.8	0.028 *	118.9 ± 20.7	112.6 ± 16.3	0.022	119.4 ± 28.9	116.4 ± 21.6	ns
	**Phase 1 Baseline**			**Phase 2 Post-Activity**			**Phase 3 Recovery**		
	R30	L30	*p*	R30	L30	*p*	R30	L30	*p*
LDF PU	13.1 ± 3.9	12.7 ± 4.8	ns	19.7 ± 5.2	19.0 ± 5.3	ns	13.4 ± 5.0	12.0 ± 4.0	ns
PSp CRBC	112.0 ± 33.2	109.0 ± 32.3	0.007 *	126.7 ± 32.5	123.1 ± 32.8	ns	111.2 ± 33.7	108.6 ± 27.4	ns

LDF PU, blood perfusion obtained from LDF (arbitrary units); PSp CRBC, concentration of red blood cells (arbitrary) obtained by polarized spectroscopy; R0 right limb at day zero, L0 left limb at day zero; R30 right limb at day thirty, L30 left limb at day thirty; ns—non-significant.

**Table 3:** Variations in current hemodynamic variables between day zero (D0) and day thirty (D30). (* Statistically compared significant differences).

	Day 0	Day 30	*p*-Value
SYS_P (mmHg)	126.5 ± 8.4	119.3 ± 7.9	0.008 *
DIAS_P (mmHg)	80.3 ± 6.4	77.9 ± 6.7	0.137
MAP (mmHg)	95.7 ± 6.8	91.7 ± 6.1	0.037 *
PR (bpm)	71.7 ± 7.4	71.0 ± 8.8	0.888

SYS_P, systolic pressure; DIAS_P, diastolic pressure, MAP, mean arterial pressure; PR, pulse rate.

## Data Availability

All data are available within the current manuscript.
